# Species conservation profile of the cave spider *Turinyphia cavernicola* (Araneae, Linyphiidae) from Terceira Island, Azores, Portugal

**DOI:** 10.3897/BDJ.4.e10274

**Published:** 2016-09-01

**Authors:** Paulo Alexandre Vieira Borges, Luis Carlos Crespo, Pedro Cardoso

**Affiliations:** ‡ CE3C – Centre for Ecology, Evolution and Environmental Changes / Azorean Biodiversity Group and Universidade dos Açores, Angra do Heroísmo, Azores, Portugal; § IUCN SSC Mid-Atlantic Islands Specialist Group, Angra do Heroísmo, Azores, Portugal; | University of Barcelona, Barcelona, Spain; ¶ IUCN SSC Spider & Scorpion Specialist Group, Helsinki, Finland; # Finnish Museum of Natural History, University of Helsinki, Helsinki, Finland

**Keywords:** Cave species, islands, IUCN, red list, tourism, troglobiont

## Species information

Scientific name: Turinyphia cavernicola

Species authority: Wunderlich, 2008

Common names: Algar do Carvão cave spider

Kingdom: Animalia

Phylum: Arthropoda

Class: Arachnida

Order: Araneae

Family: Linyphiidae

**Figure 1.**
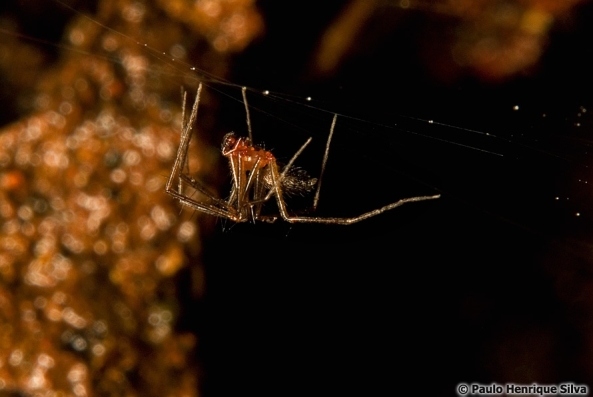
Male of *Turinyphia cavernicola* from Algar do Carvão (Terceira, Azores) (Credit: Paulo Henrique Silva).

**Figure 2.**
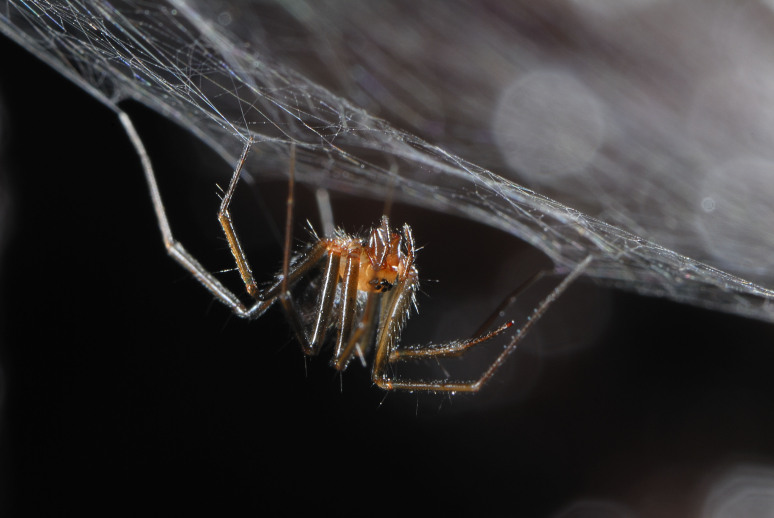
Female of *Turinyphia cavernicola* from Algar do Carvão (Terceira, Azores) (Credit: Pedro Cardoso)

Taxonomic notes: This species was described based on males only. This is a pale spider with long legs and large eyes. Male pedipalpus: tibia with single trichobothrium, paracymbium with tooth-shaped distal hook, embolus basally wide (Borges and Wunderlich 2008) (Figs 1, 2)

Region for assessment: Global

## Geographic range

Biogeographic realm: Palearctic

Countries: Portugal

**Figure 3.**
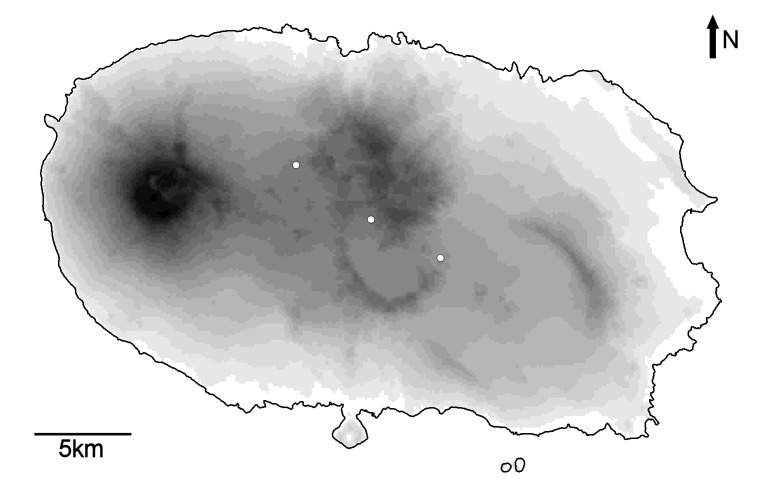
Map of Terceira (Azores, Portugal) with the three caves where the species is known to live (white dots). Darker colours represent higher altitudes.

Map of records (image): Fig. 3

Map of records (Google Earth): Suppl. material 1

Basis of EOO and AOO: Observed

**Figure 4.**
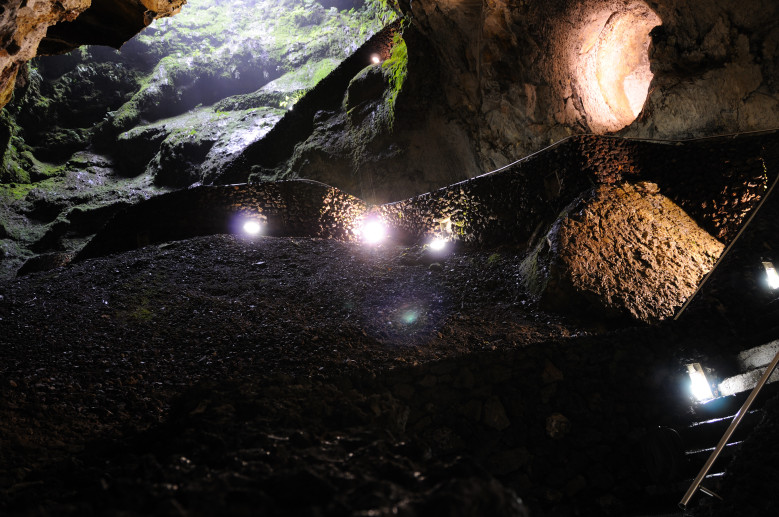
The volcanic pit Algar do Carvão (Terceira, Azores), the main location of the species *Turinyphia cavernicola*.

Basis (narrative): Relatively intensive searches have located the spider in Algar do Carvão, Gruta da Malha and Furna de Santa Maria, all in Terceira Island (Pereira et al. 2015) (Figs 3, 4)

Min Elevation/Depth (m): 460

Max Elevation/Depth (m): 583

Range description: This is a single island endemic restricted to the Island of Terceira, Azores, Portugal. The species was originally described from a single cave, the volcanic show pit Algar do Carvão, and later also found in two lava tubes: Gruta da Malha and Furna de Santa Maria, that are located nearby.

## New occurrences

### Materials

- **Type status:** Other material. **Occurrence:** recordedBy: Paulo A. V. Borges; individualCount: 1; sex: male; lifeStage: adult; **Taxon:** scientificName: Turinyphia cavernicola; kingdom: Animalia; phylum: Arthropoda; class: Arachnida; order: Araneae; family: Linyphiidae; genus: Turinyphia; specificEpithet: cavernicola; taxonRank: species; scientificNameAuthorship: Wunderlich, 2008; vernacularName: Algar do Carvăo cave spider; **Location:** islandGroup: Azores; island: Terceira; country: Portugal; stateProvince: Azores; municipality: Praia da Vitória; locality: Gruta da Malha; verbatimLatitude: 4289017; verbatimLongitude: 477951; decimalLatitude: 38.749568; decimalLongitude: -27.253739; geodeticDatum: WGS84; coordinateUncertaintyInMeters: 10; georeferenceProtocol: GPS; **Identification:** identifiedBy: Paulo A.V. Borges; **Event:** habitat: Cave; **Record Level:** institutionID: University of the Azores; collectionID: Entomoteca Dalberto Teixeira Pombo; basisOfRecord: PreservedSpecimen
- **Type status:** Other material. **Occurrence:** recordedBy: Fernando Pereira, P.A.V.Borges; individualCount: 1; sex: male; lifeStage: adult; **Taxon:** scientificName: Turinyphia cavernicola; kingdom: Animalia; phylum: Arthropoda; class: Arachnida; order: Araneae; family: Linyphiidae; genus: Turinyphia; specificEpithet: cavernicola; taxonRank: species; scientificNameAuthorship: Wunderlich, 2008; vernacularName: Algar do Carvăo cave spider; **Location:** islandGroup: Azores; island: Terceira; country: Portugal; stateProvince: Azores; municipality: Angra do Heroísmo; locality: Algar do Carvăo; verbatimLatitude: 4286675; verbatimLongitude: 481200; decimalLatitude: 38.728071; decimalLongitude: -27.215393; geodeticDatum: WGS84; coordinateUncertaintyInMeters: 10; georeferenceProtocol: GPS; **Identification:** identifiedBy: Paulo A.V. Borges; **Event:** eventDate: 1999-11-19; habitat: Cave; **Record Level:** institutionID: University of the Azores; collectionID: Entomoteca Dalberto Teixeira Pombo; basisOfRecord: PreservedSpecimen
- **Type status:** Other material. **Occurrence:** recordedBy: Isabel Amorim, Fernando Pereira; individualCount: 1; sex: male; lifeStage: adult; **Taxon:** scientificName: Turinyphia cavernicola; kingdom: Animalia; phylum: Arthropoda; class: Arachnida; order: Araneae; family: Linyphiidae; genus: Turinyphia; specificEpithet: cavernicola; taxonRank: species; scientificNameAuthorship: Wunderlich, 2008; vernacularName: Algar do Carvăo cave spider; **Location:** islandGroup: Azores; island: Terceira; country: Portugal; stateProvince: Azores; municipality: Angra do Heroísmo; locality: Furna de Santa Maria; verbatimLatitude: 4285000; verbatimLongitude: 484200; decimalLatitude: 38.713502; decimalLongitude: -27.181735; geodeticDatum: WGS84; coordinateUncertaintyInMeters: 10; georeferenceProtocol: GPS; **Identification:** identifiedBy: Paulo A.V. Borges; **Event:** habitat: Cave; **Record Level:** institutionID: University of the Azores; collectionID: Entomoteca Dalberto Teixeira Pombo; basisOfRecord: PreservedSpecimen
- **Type status:** Other material. **Occurrence:** recordedBy: Fernando Pereira, P.A.V.Borges; individualCount: 8; sex: unknown; lifeStage: Juveniles; **Taxon:** scientificName: Turinyphia cavernicola; kingdom: Animalia; phylum: Arthropoda; class: Arachnida; order: Araneae; family: Linyphiidae; genus: Turinyphia; specificEpithet: cavernicola; taxonRank: species; scientificNameAuthorship: Wunderlich, 2008; vernacularName: Algar do Carvăo cave spider; **Location:** islandGroup: Azores; island: Terceira; country: Portugal; stateProvince: Azores; municipality: Angra do Heroísmo; locality: Algar do Carvăo; verbatimLatitude: 4286675; verbatimLongitude: 481200; decimalLatitude: 38.728071; decimalLongitude: -27.215393; geodeticDatum: WGS84; coordinateUncertaintyInMeters: 10; georeferenceProtocol: GPS; **Identification:** identifiedBy: Paulo A.V. Borges; **Event:** eventDate: 1999-11-19; habitat: Cave; **Record Level:** institutionID: University of the Azores; collectionID: Entomoteca Dalberto Teixeira Pombo; basisOfRecord: PreservedSpecimen
- **Type status:** Other material. **Occurrence:** recordedBy: Fernando Pereira, P.A.V.Borges; individualCount: 1; sex: unknown; lifeStage: Juveniles; **Taxon:** scientificName: Turinyphia cavernicola; kingdom: Animalia; phylum: Arthropoda; class: Arachnida; order: Araneae; family: Linyphiidae; genus: Turinyphia; specificEpithet: cavernicola; taxonRank: species; scientificNameAuthorship: Wunderlich, 2008; vernacularName: Algar do Carvăo cave spider; **Location:** islandGroup: Azores; island: Terceira; country: Portugal; stateProvince: Azores; municipality: Angra do Heroísmo; locality: Algar do Carvăo; verbatimLatitude: 4286675; verbatimLongitude: 481200; decimalLatitude: 38.728071; decimalLongitude: -27.215393; geodeticDatum: WGS84; coordinateUncertaintyInMeters: 10; georeferenceProtocol: GPS; **Identification:** identifiedBy: Paulo A.V. Borges; **Event:** eventDate: 1999-08-10; habitat: Cave; **Record Level:** institutionID: University of the Azores; collectionID: Entomoteca Dalberto Teixeira Pombo; basisOfRecord: PreservedSpecimen
- **Type status:** Other material. **Occurrence:** recordedBy: Fernando Pereira, P.A.V.Borges; individualCount: 5; sex: unknown; lifeStage: Juveniles; **Taxon:** scientificName: Turinyphia cavernicola; kingdom: Animalia; phylum: Arthropoda; class: Arachnida; order: Araneae; family: Linyphiidae; genus: Turinyphia; specificEpithet: cavernicola; taxonRank: species; scientificNameAuthorship: Wunderlich, 2008; vernacularName: Algar do Carvăo cave spider; **Location:** islandGroup: Azores; island: Terceira; country: Portugal; stateProvince: Azores; municipality: Angra do Heroísmo; locality: Algar do Carvăo; verbatimLatitude: 4286675; verbatimLongitude: 481200; decimalLatitude: 38.728071; decimalLongitude: -27.215393; geodeticDatum: WGS84; coordinateUncertaintyInMeters: 10; georeferenceProtocol: GPS; **Identification:** identifiedBy: Paulo A.V. Borges; **Event:** eventDate: 1999-12-29; habitat: Cave; **Record Level:** institutionID: University of the Azores; collectionID: Entomoteca Dalberto Teixeira Pombo; basisOfRecord: PreservedSpecimen

## Extent of occurrence

EOO (km2): 2

Trend: Decline (inferred)

Justification for trend: The species is a specialized troglobite living in constant humidity conditions. Many caves in Terceira Island are being impacted by pollution due to the intensive cattle production in the island of Terceira, with the changes in ecological conditions of caves in the last 50 years, namely the change of the N,P abiotic cycles and changes in the water pH (Hathaway et al. 2014). The addition of fences around the cave will be an important mitigation measure.

Past decline (%): 0

Causes ceased?: No

Causes understood?: Yes

Causes reversible?: Yes

Extreme fluctuations?: No

## Area of occupancy

Trend: Decline (inferred)

Justification for trend: In Terceira Island there are 15 well-surveyed caves and we found subpopulations in only three. The trend of decline is partly based on the assumption that this species can occur in all these caves and that the absence is due not only to biological reasons (type of cave; age of the lava flow) but mainly to anthropogenic disturbance on caves during the last 50 years. Most of the caves were in the past covered by dense humid native forest, and forest clearence promoted changes in humidity and resource availability in cave environment.

Past decline (%): 20

Future decline (%): 5

Causes ceased?: No

Causes understood?: Yes

Causes reversible?: Yes

Extreme fluctuations?: No

AOO (km2): 0,75

## Locations

Number of locations: 3

Justification for number of locations: After a detailed survey of 15 caves in Terceira island the species was only found at Algar do Carvão, Gruta da Malha and Furna de Santa Maria. Each is affected by different threats, mainly touristic pressure in the first and cattle production with consequent deforestation and nutrient input into caves in the latter two.

Trend: Decline (inferred)

Justification for trend: After a detailed survey of 15 caves in Terceira island the species was only found at Algar do Carvão, Gruta da Malha and Furna de Santa Maria, which is a small number of locations for a predictably larger range (up to 5 times larger) just 50 years ago.

Extreme fluctuations?: No

## Population

Trend: Decline (inferred)

Justification for trend: Inferred from decrease in AOO and habitat quality.

Past decline (%): 20

Future decline (%) (over 10 years or 3 generations, whichever the longer): 5

Basis for decline: (c) a decline in area of occupancy, extent of occurrence and/or quality of habitat

Causes ceased?: No

Causes understood?: Yes

Causes reversible?: Yes

Extreme fluctuations?: No

Population Information (Narrative): Three subpopulations are known in the island, but two of them are very small and located in disturbed lava tubes. The single large subpopulation is located in the show cave Algar do Carvão, which is under intensive pressure due to increasing levels of visitation in the last ten years.

## Subpopulations

Number of subpopulations: 3

Trend: Decline (inferred)

Justification for trend: The species original distribution was potentially 70 km2, probably including most of the 15 caves surveyed in the Terceira Island, the current range representing a reduction of 93%. However considerable searching efforts around the current caves where the species occurs have failed to find additional subpopulations.

Extreme fluctuations?: No

Severe fragmentation?: Yes

Justification for fragmentation: The large system of lava tubes in Terceira island is fragmented both naturally and artificially. Natural fragmentation is due to the occurence of several independent historical lava-flows in the island. Artificial fragmentation is due to recent destruction of caves for road construction and intensive pasture implementation. Two out of thee subpopulations are considered non-sustainable.

## Habitat

System: Terrestrial

Habitat specialist: Yes

Habitat (narrative): The species is a troglobite specialist occuring only in humid lava tubes and volcanic pits. The sheet webs are built in small holes and crevices in the lateral walls of the caves.

Trend in extent, area or quality?: Decline (estimated)

Justification for trend: The intensive cattle production in the island of Terceira increased a lot in the last twenty years and creates high disturbance and pollution in cave systems. This might be the cause for inferred recent reduction in AOO. Touristic pressure might also be a threat in the single show-cave within its geographic range, through reduction in habitat quality.

Figure(s) or Photo(s): Fig. 4 - Algar do Carvão

### Habitat

Habitat importance: Major Importance

Habitats: 7. Caves and Subterranean Habitats (non-aquatic)

## Ecology

Size: 2 mm

Generation length (yr): 1

Dependency of single sp?: No

Ecology and traits (narrative): The species builds sheet-webs across small holes in volcanic basaltic rock. Usually occurs from twilight conditions near cave openings to deep parts of the caves (Borges and Wunderlich 2008, Martín et al. 2008, Martín et al. 2010). In the main pit-cave of Algar do Carvão the contruction of the lateral walls of the stairs with stones from the cave allowed the creation of additional supports for the webs.

## Threats

### Threats

Threat type: Ongoing

Threats: 2. Agriculture & aquaculture

## Conservation

Justification for conservation actions: An area of 40.5ha around Algar do Carvão was classified as “Regional Natural Monument” by the Regional Decree nr 9/2004/A, of March 23rd 2004, due to its unique volcanic features and its environmental importance. Since pasture intensification is one main threat, this might be important to safeguard the species survival in the future and should be extended beyond the current area, possibly allowing the recovery of other caves to original conditions where the species might be reintroduced.

### Conservation actions

Conservation action type: In Place

Conservation actions: 1. Land/water protection

### Conservation actions

Conservation action type: Needed

Conservation actions: 3. Species management

## Other

### Research needed

Research needed: 3. Monitoring

Justification for research needed: The main population in Algar do Carvão needs a long-term monitoring programme to evaluate the impact of the increasing touristic activities in this show cave.

## Supplementary Material

Supplementary material 1 Extent of Occurrence of Turinyphia cavernicola. Data type: Geographic range
File: oo_99269.kml Paulo A.V. Borges, Pedro Cardoso
